# Direct and Osmolarity-Dependent Effects of Glycine on Preimplantation Bovine Embryos

**DOI:** 10.1371/journal.pone.0159581

**Published:** 2016-07-26

**Authors:** Jason R. Herrick, Sarah M. Lyons, Alison F. Greene, Corey D. Broeckling, William B. Schoolcraft, Rebecca L. Krisher

**Affiliations:** 1 National Foundation for Fertility Research, 10290 RidgeGate Cr., Lone Tree, CO, 80124, United States of America; 2 Proteomics and Metabolomics Facility, Colorado State University, C-121 Microbiology Building, 2021 Campus Delivery, Fort Collins, CO, 80523, United States of America; 3 Colorado Center for Reproductive Medicine, 10290 RidgeGate Cr., Lone Tree, CO, 80124, United States of America; Hull York Medical School, UNITED KINGDOM

## Abstract

Concentrations of glycine (Gly) in embryo culture media are often lower (~0.1 mM) than those in oviductal or uterine fluids (≥1.2 mM). The objective of this study was to determine direct and osmolarity-dependent effects of physiological concentrations of Gly on blastocyst formation and hatching, cell allocation to the trophectoderm (TE) and inner cell mass (ICM), and metabolic activity of bovine embryos. In experiment 1, zygotes were cultured with 100 or 120 mM NaCl and 0 or 1 mM Gly for the first 72 h of culture. Blastocyst formation and hatching were improved (P<0.05) when embryos were cultured with 100 compared to 120 mM NaCl. Inclusion of 1 mM Gly improved (P<0.05) blastocyst formation compared to 0 mM Gly, but this effect was only significant (P<0.05) for embryos cultured with 120 mM NaCl, suggesting bovine embryos can utilize Gly as an osmolyte. In experiment 2, embryos were cultured with 0.1, 1.1, 2.1, or 4.1 mM Gly (100 mM NaCl) for the final 96 h of culture. Blastocyst development was not affected (P>0.05) by Gly, but hatching (0.1 mM Gly, 18.2%) was improved (P<0.05) when embryos were cultured with 1.1 (31.4%) or 2.1 (29.4%) mM Gly. Blastocyst, TE, and ICM cell numbers were not affected (P>0.05) by Gly in either experiment. Blastocysts produced alanine, glutamine, pyruvate, and urea and consumed aspartate, but this metabolic profile was not affected (P>0.05) by Gly. In conclusion, Gly (1.0 mM) improves the development of both early and late stage embryos, but beneficial effects are more pronounced for early embryos exposed to elevated osmolarity.

## Introduction

Amino acids are present in oviductal and uterine fluids [1–3] and serve a variety of physiological functions in the preimplantation embryo. Aside from being substrates for protein synthesis, amino acids are important for ATP production [4,5], purine and pyrimidine synthesis [5], methylation [6], ammonium detoxification [7,8], maintaining the REDOX balance of the cell [9], and as signaling molecules [10,11]. It is perhaps not surprising then that their inclusion in embryo culture media has profound, beneficial effects on embryonic development and viability [12–16]. As a result, some, if not all, amino acids are included in the formulations of virtually all culture media for a variety of species [12,13,16–20].

A comparison of the formulations of embryo culture media and reports on the composition of oviductal and uterine fluids indicates that cultured embryos are being exposed to non-physiological concentrations of some amino acids. Glycine (Gly) is present at ~0.05 to 0.1 mM in many embryo culture media based on the composition of Minimum Essential Medium [12,16,17,20]. However, Gly is the most abundant amino acid in reproductive tract fluids, with reports indicating physiological concentrations for bovine embryos are between 1.2 to 4.4 mM [2,21], with one report as high as 12.0 mM [22]. This discrepancy between in vivo and in vitro concentrations of Gly is particularly troubling given the direct relationship between extracellular and intracellular concentrations of Gly in embryos, the use of Gly by ICM cells, and the critical role of Gly in several aspects of cellular homeostasis and embryo development [23–25].

The most widely studied role of Gly during preimplantation development is its role in the maintenance of cell volume and intracellular osmolarity in hypertonic environments [26]. Transporters for Gly appear soon after ovulation, and mechanisms for the accumulation of Gly persist throughout preimplantation development [27–29]. However, this is not the only role of Gly. Glycine is necessary for the synthesis of purines, S-adenosylmethionine, and glutathione, [5]. Glycine is also involved in one carbon metabolism, which maintains intracellular pools of methyl donors and influences epigenetic alterations during early development [6,30].

Several studies have confirmed the importance of Gly for development of bovine embryos by showing improved development when this amino acid is added to the culture medium [31–33]. However, all of the previous studies have examined Gly supplementation through the entire culture period and have not addressed stage-specific differences between embryos before and after the maternal to zygotic transition. Similarly, none of the previous studies have addressed the potential role of Gly as an osmolyte in bovine embryos during the early cleavage stages when they are the most sensitive to environmental conditions. The objectives of this study were to evaluate the effects of Gly supplementation to the first (zygote to 8-cell, in the presence of 100 or 120 mM NaCl) and second (8-cell to hatching blastocyst) steps of a sequential media system to determine the effects on blastocyst formation, blastocyst hatching, cell allocation to the trophectoderm (TE) and inner cell mass (ICM) of resulting blastocysts, and blastocyst metabolism. Our hypothesis was that Gly would stimulate embryonic development in both stages of culture, with a more pronounced effect on early embryos cultured in a medium with increased osmolarity.

## Material and Methods

### *In Vitro* Maturation

Bovine cumulus-oocyte complexes (COCs) were collected from abattoir-derived ovaries by a commercial supplier (DeSoto Biosciences, TN, USA). Unless specified otherwise, all reagents were purchased from Sigma-Aldrich (St. Louis, MO, USA). Groups of 50 COCs were matured in 2 ml of a defined maturation medium (Table 1) containing 50 ng/ml recombinant murine EGF, 0.2 IU/ml bovine FSH (Sioux Biochemical Inc., Sioux Center, IA, USA), 0.25 mg/ml recombinant human hyaluronan (Novozymes, Bagsvaerd, Denmark), and 2.5 mg/ml recombinant human albumin (AlbIX, Novozymes) in sealed tubes gassed with 5% CO_2_ in air and maintained at ~38.5°C in a portable incubator during overnight shipment to our laboratory.

**Table 1:** Composition of Culture Media.

	IVM	IVF	IVC1	IVC2
Component (mM)	(bOMM)^1^	(bOFM)^2^	(bOEC1)^3^	(bOEC2)^3^
NaCl	100.0	100.0	100.0	100.0
KCl	5.0	5.0	5.0	5.0
KH_2_PO_4_	0.5	0.5	0.5	0.5
CaCl_2_-2H_2_O	1.7	1.7	1.7	1.7
MgSO_4_-7H_2_O	1.2	0.2	1.2	1.2
NaHCO_3_	25.0	25.0	25.0	25.0
Glucose	5.0	0.5	0.5	
Fructose				3.0
Pyruvate	0.4	0.4	0.3	0.1
L-Lactate	6.0	6.0	6.0	6.0
Citrate	0.5		0.25	0.5
Glycine^4^	2.0		**0** or 1.0	0, 1.0, **2.0**, or 4.0
GlutaMAX (Ala-Gln)^5^	1.0	1.0	1.0	1.0
Taurine	5.0	0.1	0.1	0.1
NEAA^6^	1x	1x	0.25x	1x
EAA^6^	0.5x		0.25x	0.5x
Vitamins^6^	1x			1x
myo-Inositol^7^				0.075
EDTA			0.005	
ITS (μg, μg, ng/ml)^8^	0.5, 0.26, 0.34		0.5, 0.26, 0.34	2.5, 1.38, 1.68
rHyaluronan (mg/ml)^9^	0.25		0.125	0.125
rHSA (mg/ml)^9^	2.5			
FAF BSA (mg/ml)^10^		8.0	8.0	8.0
Gentamicin (μg/ml)	50	50	25	25

^1^Bovine Oocyte Maturation Medium also contains 0.6 mM cysteine, 0.5 mM cysteamine, 50 ng/ml murine EGF, and 0.2 IU/ml bovine FSH.

^2^Bovine Optimized Fertilization Medium also contains 2.0 mM caffeine and 7.5 μg/ml heparin.

^3^Bovine Optimized Embryo Culture Medium 1 and 2

^4^Amounts listed are in addition to the Gly present in NEAA (1x = 0.1 mM). Concentrations in bold are those used for IVC1 in Experiment 2 (0.0 mM) or IVC2 of Experiment 1 (2.0 mM).

^5^From In Vitrogen (Thermo Fisher Scientific)

^6^Nonessential Amino Acids (NEAA), Essential Amino Acids (EAA), and Vitamins from cellgro (Corning, Corning, NY, USA). ‘1x’ indicates inclusion at concentrations equivalent to those found in Minimum Essential Medium (MEM).

^7^In addition to the 0.011mM present in the MEM Vitamins.

^8^Contains recombinant human insulin and transferrin (cellgro)

^9^Recombinant human hyaluronan (Hyasis) and albumin (AlbIX) from Novozymes.

^10^Fatty-Acid Free (FAF) BSA (MP Biomedicals)

### *In Vitro* Fertilization

Twenty two to twenty three hours after COCs were placed into maturation medium, COCs were recovered from the shipping tubes, washed, and transferred to 45 μl drops (10 COCs/drop under OvOil, Vitrolife, Englewood, CO, USA) of fertilization medium (Table 1) containing 2.0 mM caffeine, 7.5 μg/ml heparin, and 8.0 mg/ml fatty-acid free (FAF) bovine serum albumin (BSA, MP Biomedicals, Solon, OH, USA). Cryopreserved spermatozoa were thawed and processed by density gradient centrifugation (45%:90%, PureSperm, Nidacon, Mölndal, Sweden), followed by two washes in a MOPS-buffered medium. Spermatozoa were diluted with fertilization medium and added to drops containing COCs for a final concentration of 2 x 10^6^ spermatozoa/ml. Gametes were co-incubated in 7.5% CO_2_ in air at 38.7°C for 20–22 h. This gas concentration is increased to compensate for the elevation of our laboratory (~1830 m above sea level) and is approximately equal to 6.0% CO_2_ at sea level (media pH = 7.2 to 7.3).

### *In Vitro* Embryo Culture

Presumptive zygotes were removed from fertilization drops and denuded of remaining cumulus cells and loosely bound spermatozoa by shaking on a vortex mixer for 2.5 min. In experiment 1, zygotes were randomly allocated to a medium designed for cleavage stage bovine embryos (bOEC1, Table 1) in a 2x2 factorial experiment examining the effects of NaCl (100 or 120 mM) and Gly (0 or 1 mM added to the base medium containing 0.025 mM Gly). Increasing the concentration of NaCl from 100 mM to 120 mM increased the osmolarity of the medium from 247.9 ± 0.4 to 286.3 ± 08 mOsm, respectively. During the first 72 h of culture, embryos were cultured in groups of 10 in 20 μl drops under Ovoil. On day 3 (72 h in bOEC1, 96 h post-insemination), cleavage to at least the 2-cell stage was evaluated and embryos with more than 4-cells were washed and transferred to fresh medium designed for compaction and blastocyst formation containing 2.1 mM Gly (bOEC2, Table 1). For the final 96 h of culture, embryos were cultured in groups of 5 in 20 μl drops under Ovoil. In experiment 2, all zygotes were cultured in the control medium from experiment 1 (bOEC1; 100 mM NaCl and 0.025 mM Gly) for 72 h. On day 3, embryos (>4-cells) were randomly allocated to one of four media containing 100 mM NaCl and 0.1, 1.1, 2.1, or 4.1 mM Gly (263.6 ± 1.2 mOsm). On day 7 of culture (96 h in bOEC2, 192 h post-insemination), blastocyst formation and hatching were evaluated. All cultures were conducted in 7.5% CO_2_ and 6.5% O_2_ (also increased due to elevation) at 38.7°C.

### Determination of Trophectoderm and Inner Cell Mass Cell Numbers

**Fig 1:**
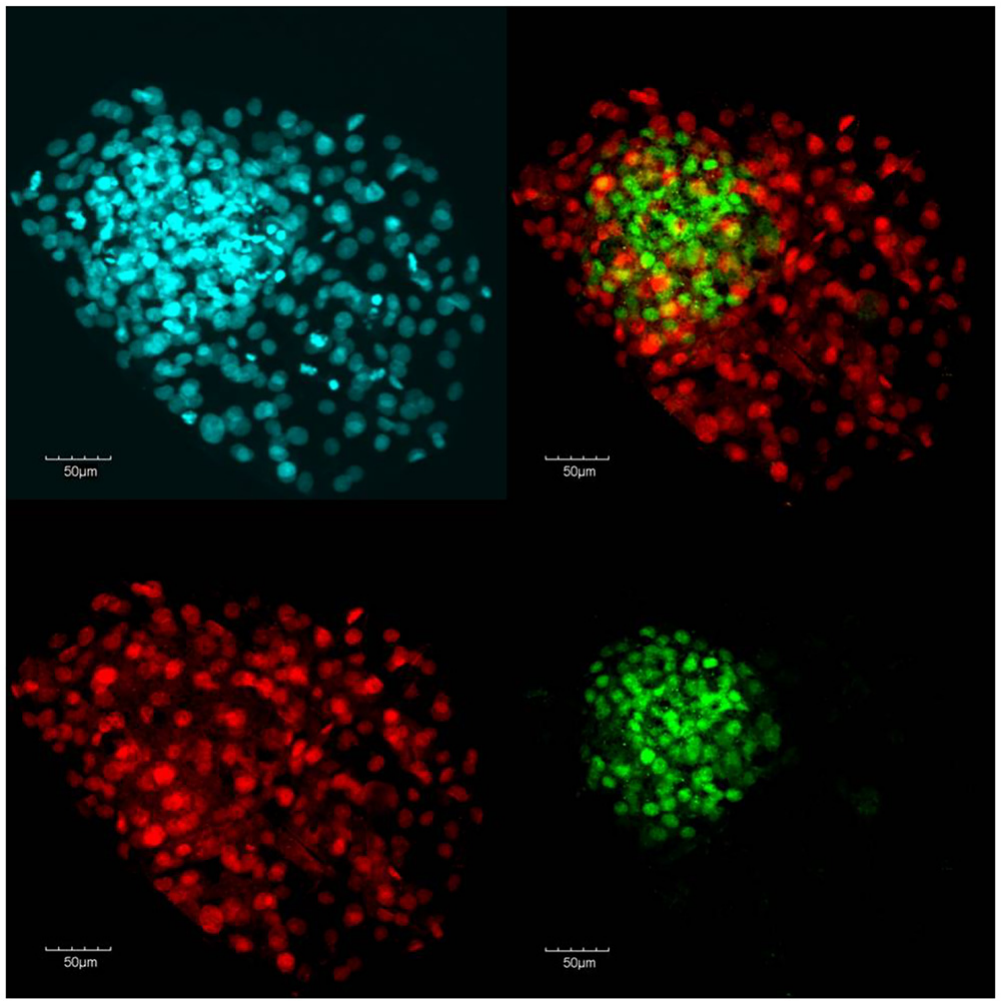
Hatching bovine blastocyst stained with DAPI (4′,6-Diamidine-2′-phenylindole dihydrochloride; upper left) and labelled with antibodies for CDX2 (trophectoderm-specific, red, lower left) and SOX2 (inner cell mass-specific, green, lower right).

Hatching and fully-hatched blastocysts were fixed for 20 min in 4% paraformaldehyde (Electron Microscopy Sciences, Hatfield, PA, USA) and then stored in PBS with 0.5% BSA (MP Biomedicals) until staining. Blastocysts were washed three times in PBS with 0.1% PVP and 0.1% Triton X-100 (TX100) and then permeabilized in PBS with 1.0% TX100 (30 min). After blocking (2 h) in PBS with 0.1% TX100, 0.1 M glycine, 0.5% BSA, and 10% (v/v) horse serum, blastocysts were incubated with primary antibodies (18 to 24 h, 4°C) for SOX2 (Biogenex, Fremont, CA, rabbit monoclonal, anti-human; AN579) and CDX2 (Biogenex, mouse monoclonal, anti-human; MU392A) [34,35]. Following three washes in PBS with 0.1% PVP and 0.1% TX100, blastocysts were incubated (1 h) with secondary antibodies (Alexa Fluor 488 donkey anti-rabbit IgG (A-2126, SOX2) and Alexa Fluor 555 goat anti-mouse IgG (A-21424, CDX2; Invitrogen, Thermo Fisher Scientific, Waltham, MA, USA). Blastocysts were washed three times and mounted on a glass slide in ProLong Gold Antifade reagent containing DAPI (Life Technologies, Thermo Fisher Scientific). Cells were visualized using a fluorescent microscopy (Olympus BX52) and counted using the manual count function of MetaMorph software. Cells positive for SOX2 were considered ICM cells and cells positive for CDX2 were considered TE cells (Fig 1). The total number of cells in the blastocyst was calculated as the sum of SOX2- and CDX2-positive cells.

### Assessment of Blastocyst Metabolism

After 48 h of culture in bOEC2 (120 h of culture, 144 h post insemination), embryos were transferred to fresh medium in wells of a 60-well culture dish (1 embryo in 12 μl/well, Nunc Mini Tray 163118, Thermo Fisher Scientific) covered with Ovoil. Gly concentrations in the bOEC2 were identical to those used for group culture during the previous 48 h (2.1 mM for all embryos in experiment 1 or 0.1, 1.1, 2.1, or 4.1 mM in experiment 2). Embryos were then cultured (7.5% CO_2_, 6.5% O_2_, 38.7°C) for an additional 48 h (96 h total in bOEC2) and evaluated for blastocyst formation and hatching on day 7 (192 h post-insemination). Medium (10 μl) was collected from wells that contained embryos that developed to the hatching or fully hatched blastocyst stage. Control medium was also collected from wells in the same dish that did not contain an embryo. Immediately after collection, medium samples were frozen in liquid nitrogen and stored at -80°C until analysis.

Concentrations of nutrients were determined using gas chromatography (GC) and mass spectrometry (MS) as previously described [36]. Aliquots (6 μl) of the collected media were dried using a Savant AES 2010 speedvac system (ThermoSavant, Waltham, MA, USA) and then methoximated by incubating (45 min, 60°C) with 12.5 μl of methoxyamine HCL (25 mg/ml) in pyridine. Following sonication for 10 min and an additional 45 min at 60°C, 12.5 μl of N-methyl-N-trimethylsilyltrifluoroacetamide (MSTFA) with 1% trimethylchlorosilane (TMCS, Thermo Fisher Scientific) was added. After incubation for 30 min at 60°C, samples were centrifuged (3000g, 5 min at 4°C), and cooled to room temperature. Twenty μl of the resulting supernatant were transferred to a 150 μl glass insert in a GC-MS autosampler vial for analysis using a Trace GC Ultra coupled to a Thermo ISQ mass spectrometer (Thermo Fisher Scientific). Aliquots (1 μl) of each sample were injected in a 1:10 split ratio twice in discrete randomized blocks (n = 2 injections/sample). Separation occurred using a 30-m TG-5MS column (0.25 mm i.d. and 0.25 μm film thickness; Thermos Scientific) with a 1.2 ml/min flow rate of helium gas and a program consisting of 80°C for 30 s, a ramp of 15°C/min to 330°C, and an 8 min hold. Masses between 50 and 650 *m/z* were scanned at five scans per second after electron impact ionization.

Raw data files were converted to.cdf format of relative quantitation. A matrix of molecular features defined by retention time and mass (*m/z*) was generated using XCMS software in R [37] feature detection and alignment. Raw peak values were normalized against total ion signal in R, outliers were detected based on total signal and principal component 1 of principle component analysis, and the mean area of the chromatographic peak was calculated among replicate injections (n = 2). Features were grouped with RAMclustR [38] and metabolites were annotated by matching retention index and mass spectra using NIST v11 and v12, Golm, Metlin, and Massbank metabolite databases. Initial experiments indicated that glutamine in the samples was converted to pyroglutamate during sample preparation and/or analysis [36]. Therefore, pyroglutamate concentrations are considered to represent the concentration of glutamine in the samples.

### Statistical Analysis

Embryonic development was analyzed using the generalized linear mixed model (GLIMMIX) procedure in SAS. The proportions of embryos developing to the blastocyst or hatching blastocyst stage was based on the number of zygotes placed into culture (experiment 1) or the number of embryos placed into the second stage culture medium (>4-cells at 72 h; experiments 1 and 2). Each embryo was scored as a 1 or 0 depending on whether or not it achieved the desired stage of development (e.g., cleaved, blastocyst, or hatching blastocyst) and analyzed using a binomial error distribution and a probit link function. In experiment 1, the main effects of the concentrations of NaCl and Gly and the interaction between these factors were included as fixed factors for analysis of embryonic development. In experiment 2, the concentration of Gly (0.1, 1.1, 2.1, and 4.1) was the only fixed factor for analysis of both embryonic development and blastocyst cell numbers. Replicate was included as a random factor for analysis of embryonic development.

Blastocyst cell numbers were analyzed using the mixed model procedures in SAS. In experiment 1, there were not enough hatching or fully-hatched blastocysts from the 120 mM NaCl treatments for inclusion in the analysis of blastocyst cell numbers, so the concentration of Gly was the only fixed factor. The concentration of Gly was also the only fixed factor in the second experiment.

For blastocyst metabolism, two analyses were performed in the MIXED procedure of SAS. In the first, metabolite concentrations in medium that contained an embryo were compared to those in control medium that did not contain an embryo to determine if each metabolite was significantly consumed (lower than the control) or produced (higher than the control) by the embryo during culture. Comparisons were only made between treatment and the control. In the second, metabolite concentrations for the treatments were normalized to the concentrations in the control medium (no embryo) to determine the fold-change of each metabolite relative to the control, which facilitated comparisons between treatments.

In all analyses, pairwise comparisons were made using Fisher’s protected least significant difference test and P<0.05 was considered a significant difference. All means are presented ± SEM.

## Results

### Experiment 1: Embryonic Development

**Fig 2:**
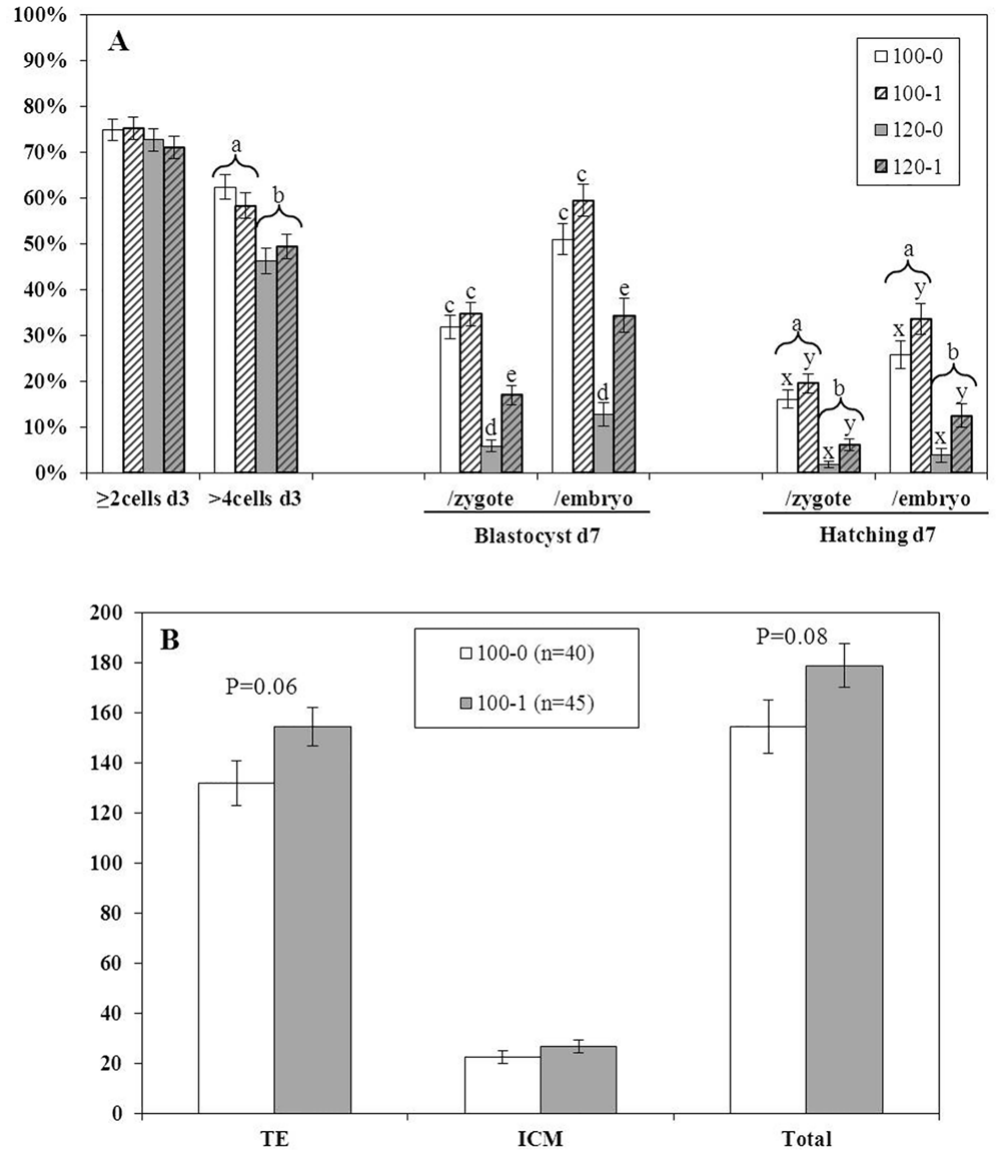
Embryonic development (A) and blastocysts cell numbers (B) for embryos cultured with 100 or 120 mM NaCl and 0 or 1 mM Gly for the first 72 h of culture (zygote to 8-cell). The number of cells in the trophectoderm (TE) and inner cell mass (ICM) and the total number of cells were determined in hatching or hatched blastocysts. Different superscripts indicate a significant (P<0.05) effect of NaCl (^a,b^), Gly (^x,y^), or the interaction between NaCl and Gly (^c,d,e^). If no superscripts are present, treatments were not different (P>0.05).

Four replicates were performed using a total of 1357 presumptive zygotes (337–342 per treatment). The proportion of zygotes that cleaved (≥71.1%) was not affected (P>0.05) by the concentration of NaCl, Gly, or the interaction between these two factors (Fig 2A). However, the proportion of embryos with >4-cells on day 3 that were moved to the second step culture medium was higher (P<0.05) for oocytes cultured with 100 mM NaCl (0 mM Gly, 62.3 ± 2.6%; 1 mM Gly, 58.3 ± 2.7%) compared to those cultured in 120 mM NaCl (0 mM Gly, 46.2 ± 2.7%; 1 mM Gly, 49.4 ± 2.7%) (Fig 2A). The main effects of the concentration of NaCl (100 >120 mM) and the concentration of Gly (1>0 mM), as well as the interaction between these two factors, significantly affected (P<0.05) blastocyst formation (per zygote and per embryo >4 cells placed into IVC2). More (P<0.05) blastocysts formed when zygotes were cultured for the first 72 h in medium containing 100 mM NaCl and 1 mM Gly (34.6 ± 2.6% of zygotes, 59.4 ± 3.5% of embryos) compared to 120 mM NaCl with either 0 (5.9 ± 1.3% of zygotes, 12.7 ± 2.7% of embryos) or 1 (17.0 ± 2.0% of zygotes, 34.3 ± 3.7%of embryos) mM Gly, but not compared to medium with 100 mM NaCl and 0 mM Gly (31.8 ± 2.5% zygotes, P = 0.43; 51.0 ± 3.5% of embryos, P = 0.09) (Fig 2A). Inclusion of 1 mM Gly in medium with 120 mM NaCl significantly improved (P<0.05) blastocyst development compared to medium with 120 mM NaCl and 0 mM Gly. The proportions of zygotes or embryos that initiated hatching were also improved (P<0.05) when embryos were cultured for the first 72 hours with reduced concentrations of NaCl (100>120 mM) and/or increased concentrations of Gly (1>0 mM), but the interaction between these factors was not significant (P>0.05, Fig 2A).

Embryos that were cultured for the first 72 hours with 100 mM NaCl and 1 mM Gly produced blastocysts that tended to have more TE cells (154.3 ± 7.8; P = 0.06) and total (178.6 ± 8.7; P = 0.08) cells than embryos cultured with 100 mM NaCl and 0 mM Gly (131.8 ± 9.0 TE, 154.3 ± 10.7 total) (Fig 2B). In the presence of 100 mM NaCl, the concentration of Gly did not affect (P>0.10) the number of ICM cells (0 mM Gly, 22.5 ± 2.7 and 1 mM Gly, 26.8 ± 2.5).

### Experiment 1: Blastocyst Metabolism

**Fig 3:**
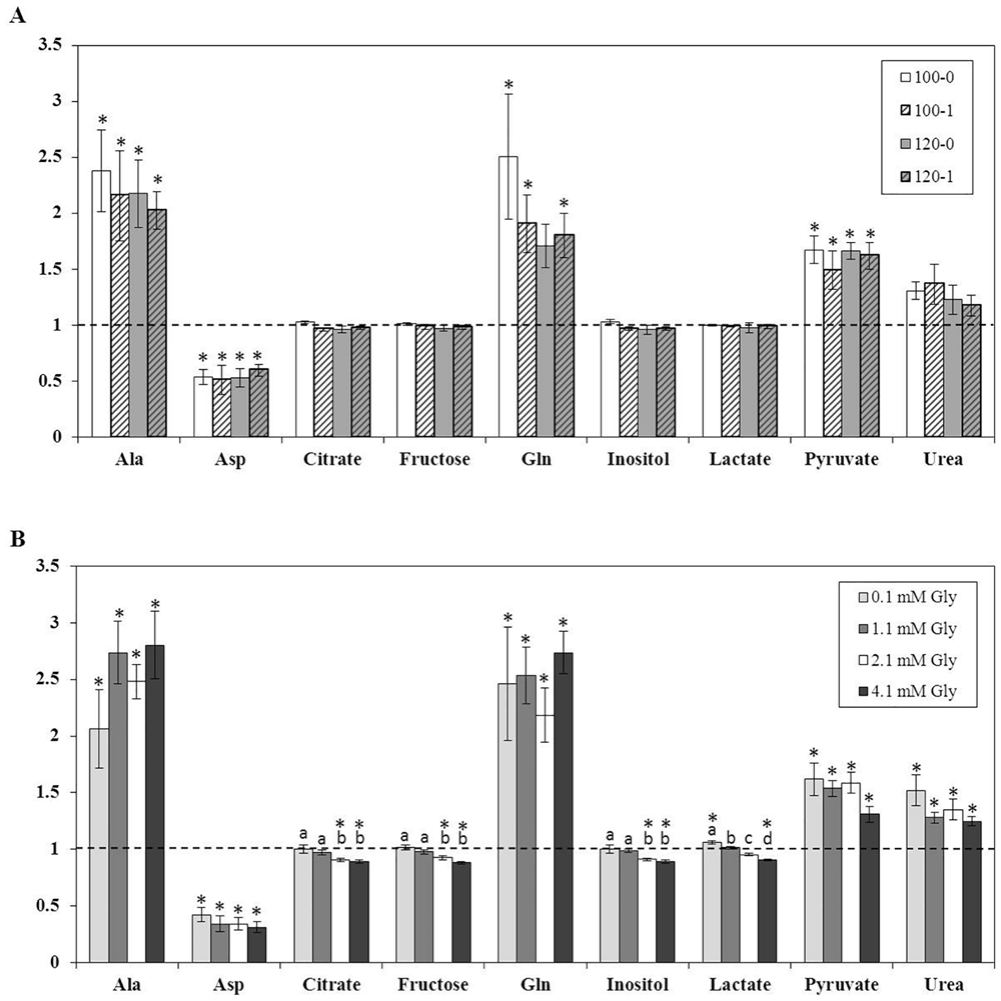
Relative change in the composition of culture media following a 48 h incubation with embryos while they developed from the morula to hatching blastocyst stage in experiment 1 (A) or 2 (B). The amount of each nutrient present in the medium at the end of the incubation is expressed as the fold change in abundance compared to control media that did not contain an embryo (dashed line). An asterisk (*) indicates a significant (P<0.05) change compared to control media. Different letters (^a,b,c,d^) indicate a significant (P<0.05) difference in the amount of each nutrient between treatments. If no asterisk or letters are present, there were no differences (P>0.05).

Media from 6 blastocysts per treatment was analyzed for 20 metabolites. Concentrations of citrate, fructose, glycine, isoleucine, lactate, leucine, lysine, methionine, inositol, ornithine, phenylalanine, proline, pyruvate, serine, threonine, and urea in the medium were not altered (P>0.05) by the embryo regardless of treatment. In all treatments, blastocysts consumed (P<0.05) aspartate (Asp) and secreted (P<0.05) alanine (Ala) and pyruvate into the medium (Fig 3A). Blastocysts from all treatments also secreted glutamine (Gln) into the medium, but this was only significant (P<0.05) for embryos that had been cultured in 100 mM NaCl with either 0 or 1 mM Gly and 120 mM NaCl with 1 mM Gly for the first 72 hours (Fig 3A). Embryos that had been cultured in 120 mM NaCl and 0 mM Gly tended (P = 0.08) to produce Gln.

### Experiment 2: Embryonic Development

**Fig 4:**
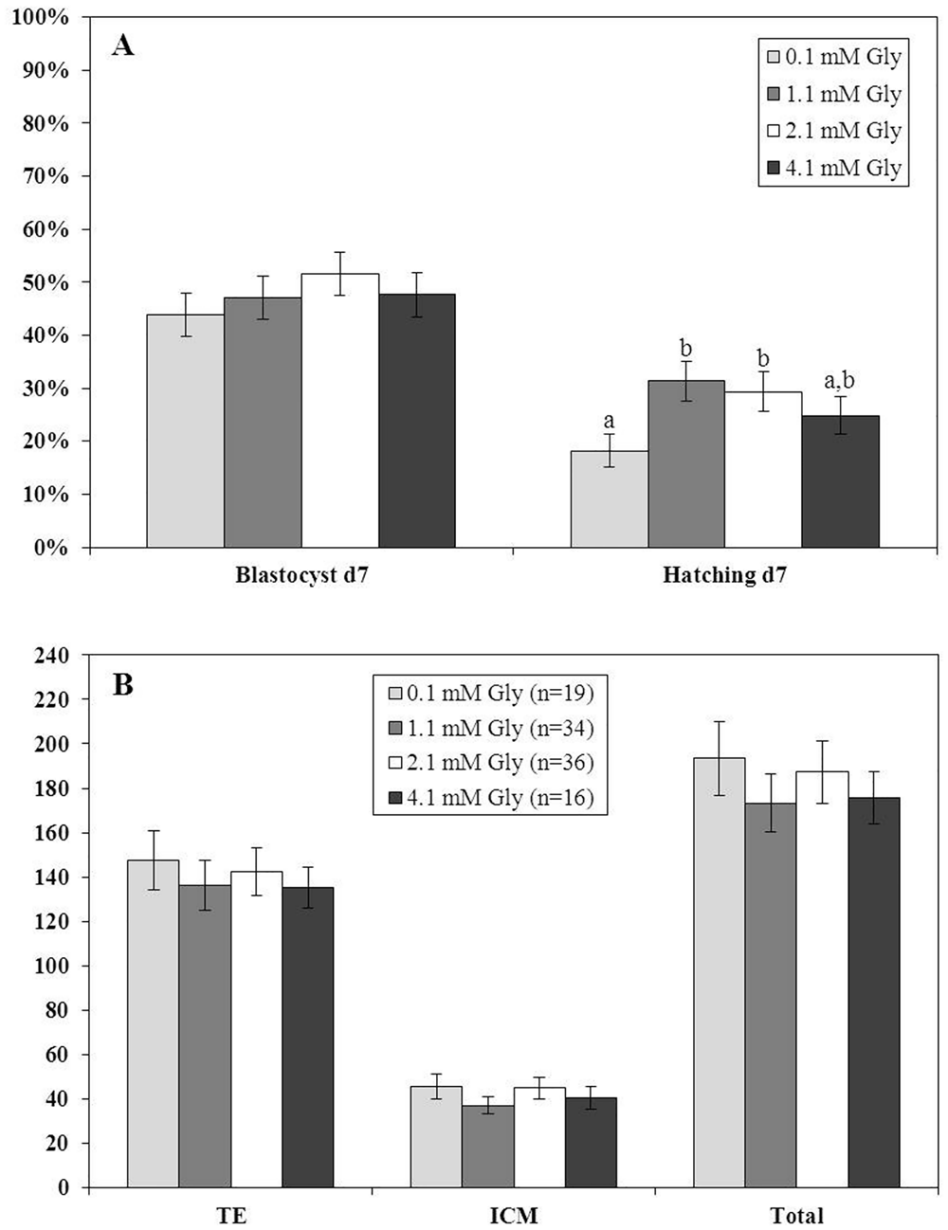
Embryonic development (per embryo >4 cells on day 3, A) and blastocysts cell numbers (B) for embryos cultured with 0.1, 1.1, 2.1, or 4.1 mM Gly for the final 96 h of culture (8-cell to hatching). The number of cells in the trophectoderm (TE) and inner cell mass (ICM) and the total number of cells were determined in hatching or hatched blastocysts. ^a,b^ Different letters indicate a significant (P<0.05) difference between treatments. If no superscripts are present, treatments were not different (P>0.05).

Four replicates were performed using a total of 603 embryos (>4-cell) with 148 to 153 cultured per treatment. The proportion of embryos developing to the blastocyst stage was not affected (P>0.05) by the concentration of Gly in the medium (43.9 ± 4.1% to 51.6 ± 4.1%) (Fig 4A). However, the proportion of cultured embryos that initiated hatching was increased (P<0.05) when embryos were cultured with 1.1 (31.4 ± 3.8%) or 2.1 (29.4 ± 3.7%) mM Gly compared to 0.1 (18.2 ± 3.2%) mM Gly (Fig 4A).

The concentration of Gly in the medium did not affect (P>0.05) the number of TE (135.3 ± 9.1 to 147.5 ± 13.3) or ICM (37.0 ± 3.8 to 45.5 ± 5.6) cells, or total the total number of cells (173.3 ± 13.2 to 193.4 ± 16.5) in the resulting blastocysts (Fig 4B).

### Experiment 2: Blastocyst Metabolism

Medium from 5 to 6 blastocysts per treatment was analyzed for 19 metabolites. Concentrations of isoleucine, leucine, lysine, methionine, ornithine, phenylalanine, proline, serine, threonine, and tyrosine in the medium were not significantly altered (P>0.05) by the embryo regardless of treatment. Blastocysts from all treatments consumed (P<0.05) Asp (Fig 3B). Blastocyst also consumed citrate, fructose, and inositol, but the amount consumed was only different (P<0.05) from the control medium when 2.1 or 4.1 mM Gly were present in the culture medium (Fig 4B). Ala, Gln, pyruvate, and urea were secreted (P<0.05) into the medium by blastocysts in all treatments, but the amount secreted was not affected (P>0.05) by the concentration of Gly (Fig 3B). Lactate was secreted (P<0.05) in the presence of 0.1 mM Gly and consumed when the concentration of Gly was increased to 2.1 (P = 0.08) or 4.1 (P<0.05) mM (Fig 3B).

## Discussion

This is the first study to investigate the stage-specific effects of physiological concentrations of Gly on the development of bovine embryos. The results indicate beneficial effects of Gly on embryonic development when present in both the early (zygote to 8-cell) or late (8-cell to blastocyst) stages of culture. Glycine could partially mitigate the negative effects of elevated NaCl/osmolarity, suggesting bovine embryos can use this amino acid as an osmolyte. Despite the beneficial effects on embryonic development, Gly had surprisingly little effect on the metabolic profile of blastocysts. However, several notable aspects of embryo metabolism were evident, providing new insight on the physiology of the bovine embryo.

Increased NaCl and medium osmolarity dramatically inhibited the development of bovine embryos, which is consistent with data from other species [18,39,40] and other studies of bovine embryos [41]. The primary difference between species seems to be the ability of the embryo to use certain amino acids as osmolytes and maintain developmental competence in media with increased osmolarity. Early stage (zygotes and 2-cell) murine embryos can use a number of different amino acids as osmolytes, with Gln, Gly, and betaine being three of the most effective [27,40]. Supplementation of culture media with any one of these amino acids can restore development to rates observed in media with lower osmolarity. Our results suggest that the bovine embryo has a limited ability to utilize Gln and Gly as osmolytes compared to murine embryos. Increasing the concentration of NaCl from 100 to 120 mM significantly reduced embryonic development even though 1.0 mM Gln was present in the medium. When Gly (1.0 mM) was added to the medium with increased NaCl, development was improved, but was still significantly lower than what was observed in the medium containing 100 mm NaCl. Similarly, Liu and Foote [41] demonstrated that bovine embryos were unable to effectively use betaine as an osmolyte when cultured in a medium with increased osmolarity. It is possible that embryonic development would have been even lower in the complete absence of any osmolytes and the low incidence of blastocyst formation observed represents the maximal ability of bovine embryos to use Gln, Gly, or any of the other amino acids present, as osmolytes. Whether the bovine embryo is uniquely sensitive to medium osmolarity and/or has a limited ability to use amino acids as osmolytes will require additional research.

Beneficial effects of increasing the concentration of Gly in the medium during the entire preimplantation culture period have been previously reported, but the concentrations of Gly required to elicit a beneficial effect on embryonic development (5 to 10 mM) in these studies [31–33] were much higher than those used in the present study (≤4.1 mM). Several of the studies in which 5 to 10 mM Gly were evaluated used PVA as the macromolecule in the medium, rather than albumin [32,33,42]. Biggers et al. [43] demonstrated a more pronounced effect of amino acid supplementation on embryo development when the medium was devoid of protein. Embryos may be able to endocytose albumin and use it as a source of amino acids [44,45]. Alternatively, the beneficial effects of Gly on the development of porcine blastocysts have been shown to be affected by the concentration of glucose in the medium [46]. The concentration of glucose in previous studies of bovine embryos was not always specified, but appears to be lower (0.0 to 0.2 mM) [31,42] than the concentration of fructose used in the present study (3.0 mM). Therefore, differences in the concentrations of Gly required to elicit a beneficial effect on embryonic development between the current study and previous studies may have been due to differences in the type of macromolecule used or the concentration of glucose present in the culture medium. It should also be noted that supraphysiological concentrations of Gly (~10 mM) can improve preimplantation development of porcine embryos in vitro, but still compromise fetal development following embryo transfer [47]. Although we are not aware of studies that have transferred bovine embryos following culture with elevated Gly, these recent findings from porcine embryos caution against the use of such high concentrations of Gly and highlight the importance of transfer experiments in assessing embryo viability.

Although supplementing the first or second step culture medium with Gly improved embryonic development, we did not observe significant consumption of this amino acid by the blastocysts. While this was unexpected, it is consistent with other reports of amino acid metabolism by bovine blastocysts in which limited (nonsignificant) consumption [48] or production [7,45,49] of Gly was reported. This discrepancy may be due, in part, to differences between statistically significant and biologically significant quantities of Gly relative to the amount present in the medium. For example, a 12 μl drop of medium containing 2.1 mM Gly contains 25,200 pmol Gly. If bovine blastocysts consume ~2 pmol of Gly per hr [48], ~96 pmol of Gly, or ~0.4% of the total Gly present, would be consumed during a 48 h incubation. While this may be a biologically significant quantity of Gly for the embryo, it is a statistically insignificant proportion of what is present in the medium. However, this does not explain why development was improved in the presence of 1.1 or 2.1 mM (22,000 or 42,000 pmol in 20 μl culture drops) Gly compared to 0.1 mM (2000 pmol) Gly if the embryo only needs 96 pmol. Van Winkle and Dickinson [23] showed that the intracellular concentration of Gly was dependent on the concentrations of other amino acids (taurine, Ala, Gln, and Glu) in the medium. A large excess of extracellular Gly may be necessary to elicit a small increase in the intracellular concentration of Gly in bovine embryos when all the amino acids are present.

The only nutrient that was consumed in significant quantities by blastocysts in all treatments in both experiments was Asp. Similar observations have been reported in studies of bovine [7,24,45,48] and murine [8,50] embryos. Other studies of bovine [49], human [51], and porcine [52] blastocysts did not observe significant consumption of Asp, but all three of these studies used much lower concentrations of Asp (0.03 mM) [53] than in the present study (0.1 mM Asp) or those reported in the bovine uterus (0.096 to 1.75 mM) [2,21,22]. Asp is a critical substrate for the maintenance of NAD^+^/NADH balance and glycolytic activity within the embryo, due to its role in the Malate-Aspartate Shuttle (MAS) [9,54]. Consumption of more than 50% of the Asp provided to the embryo in the medium may be necessary to maintain a suitable intracellular concentration for continuous activity of the MAS.

While nutrient consumption was largely limited to Asp, blastocysts produced significant quantities of Ala and Gln in both experiments, and a small amount of urea in the second. Production of Ala has been reported in multiple studies of amino acid metabolism by bovine [7,21,24,45,48,49], human [51], murine [50], and porcine [52] blastocysts. Production of Ala from pyruvate and Glu is one of the ways embryos cope with intracellular ammonium, which is toxic [7,8,17]. Secretion of Gln may serve a similar function in both murine [8] and bovine [7] blastocysts. To our knowledge this is the first report of urea production by preimplantation embryos. Orsi and Leese [7] were unable to detect urea even when 30 blastocysts were cultured in 15 μl of media for 30 h. It is possible that detection of urea may be due to the increased sensitivity of GC-MS analysis. Alternatively, disposal of ammonium via the production of urea may be associated with embryo quality, since it was only detected in our second experiment, in which ICM cell numbers were nearly double those observed in experiment 1.

Metabolic profiles also indicated significant production of pyruvate. Although this was consistent between both experiments, as well as our previous work [36], it is in stark contrast to all other studies of bovine embryos in which pyruvate consumption has been reported [24,55–57]. However, all of these studies have utilized glucose in the medium, rather than fructose [58–60]. Our previous work with bovine embryos indicates that production of pyruvate may be associated with the presence of fructose in the medium, since pyruvate production was not observed when bovine blastocysts were cultured with glucose [36]. In some tissues, fructose can enter glycolysis via fructokinase, which is downstream of hexokinase and phosphofructokinase, allowing rapid conversion to pyruvate or lactate [61,62]. Alternatively, some hexokinases react more strongly with fructose than glucose [63]. Although the existence of fructokinase and the relative affinity of hexokinases for fructose are not known for bovine blastocysts, the presence of one or more of these pathways would potentially explain the increased production of pyruvate in the presence of fructose.

The results of the present study highlight the importance of Gly on bovine embryo development, and emphasize the importance of evaluating individual amino acids during specific periods of preimplantation development. Not only did an increased (1.0 mM) concentration of Gly improve blastocysts formation and hatching, but many of the changes observed in the composition of the culture medium following embryo culture suggest active amino acid metabolism. Three (Ala, Gln, urea) of the four substrates that were produced by the embryos are involved in detoxification of ammonium that is produced when amino acids are used as a source of carbon [7,8]. Blastocysts also consumed approximately half of the available Asp, which is critical for the activity of the MAS and the maintenance of the NAD^+^/NADH ratio in the cytoplasm [9,54]. While numerous studies have evaluated the effects of amino acids on bovine embryos, few of these studies have assessed the effects of individual amino acids [13,46,64,65]. Gly is just one of many amino acids for which the concentrations used in embryo culture media are different from physiological concentrations in the oviduct and uterus. The results of the present study would suggest that additional experiments with physiological concentrations of individual amino acids may lead to the development of culture medium that better support the in vitro development of bovine embryos.
